# Predictors of Hospital Admission and Cardiac Diagnosis in Children Presenting with Chest Pain: The Role of Recurrent Presentation and Inflammatory Markers

**DOI:** 10.3390/medicina62091809

**Published:** 2026-09-19

**Authors:** Şule Demir, Murat Ayar, Ayşe Nilsu Doğan, Aykut Çağlar

**Affiliations:** 1 Division of Pediatric Emergency Medicine, Department of Pediatrics, Faculty of Medicine, Aydın Adnan Menderes University, 09010 Aydın, Türkiye; aykutcaglar@gmail.com; 2 Department of Pediatrics, Faculty of Medicine, Aydın Adnan Menderes University, 09010 Aydın, Türkiye; muratayar45@gmail.com (M.A.); nilsudogann@gmail.com (A.N.D.)

**Keywords:** chest pain, child, C-reactive protein, emergency department, recurrent presentation, risk stratification

## Abstract

*Background and Objectives*: Pediatric chest pain is usually benign, but identifying children who require admission or have a cardiac diagnosis remains challenging. We aimed to identify factors associated with these outcomes, with a focus on recurrent presentation and the combined value of C-reactive protein (CRP) and predefined red-flag findings. *Materials and Methods*: This retrospective cohort included children aged 0–18 years presenting with chest pain to a tertiary pediatric emergency department between January 2021 and January 2026; visits with insufficient documentation were excluded. Red-flag findings were predefined as effort-related chest pain, syncope, palpitations, dyspnea, fever, or a family history of sudden cardiac death. Independent predictors were assessed using multivariable Firth logistic regression, and cluster-robust standard errors were used in a sensitivity analysis to account for recurrent visits. *Results*: Of 1703 visits (1435 patients), 452 (26.5%) were recurrent presentations. Admission occurred in 64 visits (3.8%), and 91 (5.3%) had a cardiac diagnosis. Red-flag findings (OR, 5.15), abnormal ECG (OR, 4.00), CRP > 5 mg/L (OR, 3.71), male sex (OR, 2.13), and recurrent presentation (OR, 2.51) were independently associated with admission (all *p* ≤ 0.009). Recurrent presentation was not associated with a cardiac or psychogenic diagnosis. Admission and cardiac diagnosis rates increased from 1.4%/2.2% in children with neither elevated CRP nor red-flag findings to 22.2%/24.8% in those with both. Routine ECG, troponin, and chest radiography had low diagnostic yields (2.1–6.0%), whereas echocardiography performed selectively yielded abnormal findings in 79.7%. *Conclusions*: Red-flag findings, abnormal ECG, elevated CRP, and recurrence were associated with admission, whereas recurrence was not associated with diagnosis. Combining CRP with red flags may improve risk stratification, though prospective validation is needed.

## 1. Introduction

Chest pain is among the most common reasons for pediatric emergency department (ED) visits and a frequent source of anxiety for patients and families, largely because of its perceived association with cardiac disease [1,2]. In adults, chest pain is a well-established marker of life-threatening cardiac and vascular emergencies; in children, however, the underlying etiology is overwhelmingly benign, with musculoskeletal, respiratory, gastrointestinal, and psychogenic causes accounting for the vast majority of presentations, and cardiac disease identified in only a small minority [1,2,3,4].

Despite reassuring epidemiologic data, the possibility of an underlying cardiac cause continues to drive extensive diagnostic evaluation—electrocardiography, troponin measurement, chest radiography, and echocardiography—as well as hospital admissions and cardiology referrals [4,5]. Recent evidence in adults suggests that high-sensitivity cardiac troponin may reflect subclinical cardiovascular alterations even in the absence of acute illness, with associations reported across measures of cardiac function, subclinical atherosclerosis, and arterial stiffness [6]. However, whether diagnostic yield is proportional to this intensity has not been systematically quantified in unselected pediatric ED populations. This tension between low pretest probability and the imperative not to miss serious disease has prompted efforts to identify clinical features that reliably distinguish children who require further evaluation from those who can be safely discharged [4,7]. Exertional chest pain, syncope, palpitations, abnormal ECG findings, and a family history of sudden cardiac death have consistently been identified as “red-flag” findings for cardiac disease in children [7,8]. However, their diagnostic performance remains imperfect [8]. Inflammatory and hematologic markers such as C-reactive protein (CRP), the neutrophil-to-lymphocyte ratio (NLR), and the platelet-to-lymphocyte ratio (PLR) have gained attention as inexpensive adjuncts for risk stratification in other pediatric conditions [9,10], but their added value beyond clinical red-flag features in chest pain remains poorly characterized.

A further, less well-characterized dimension of pediatric chest pain is its tendency to recur: repeat ED presentations have been associated with subsequent hospital readmission and school absenteeism [11], and a recognizable subgroup of children accounts for a disproportionate share of ED visits, reflecting chronic disease burden, referral patterns, care-seeking behavior, and clinical acuity [12]. Whether recurrent presentation for chest pain is independently associated with hospital admission or with a subsequent cardiac or psychogenic diagnosis, beyond what is captured by established red-flag features, has not been specifically examined.

Therefore, to our knowledge, this study had two primary aims that have not previously been addressed together in a pediatric chest-pain population: to determine whether recurrent presentation is independently associated with hospital admission or with a subsequent cardiac or psychogenic diagnosis, and to evaluate the combined value of CRP and clinical red-flag findings for early risk stratification. As secondary, confirmatory aims, we characterized the etiologies of chest pain, quantified the utilization and diagnostic yield of commonly performed ancillary tests, and identified additional independent clinical and laboratory predictors of hospital admission and of a cardiac diagnosis.

## 2. Materials and Methods

### 2.1. Study Design, Setting, and Population

This retrospective cohort study was conducted at the Pediatric Emergency Department of Aydın Adnan Menderes University Faculty of Medicine Hospital, Aydın, Türkiye, a tertiary referral center. All consecutive patients aged 0–18 years presenting with chest pain from 1 January 2021 to 1 January 2026 were identified using the Hospital Information Management System. The inclusion criteria were: (1) age 0–18 years; (2) presentation to the pediatric ED with chest pain; and (3) presentation during the study period (1 January 2021 to 1 January 2026). The exclusion criterion was insufficient documentation to determine study eligibility or outcomes. Trauma-related chest pain and pre-existing cardiac disease were not exclusion criteria.

**Figure 1:**
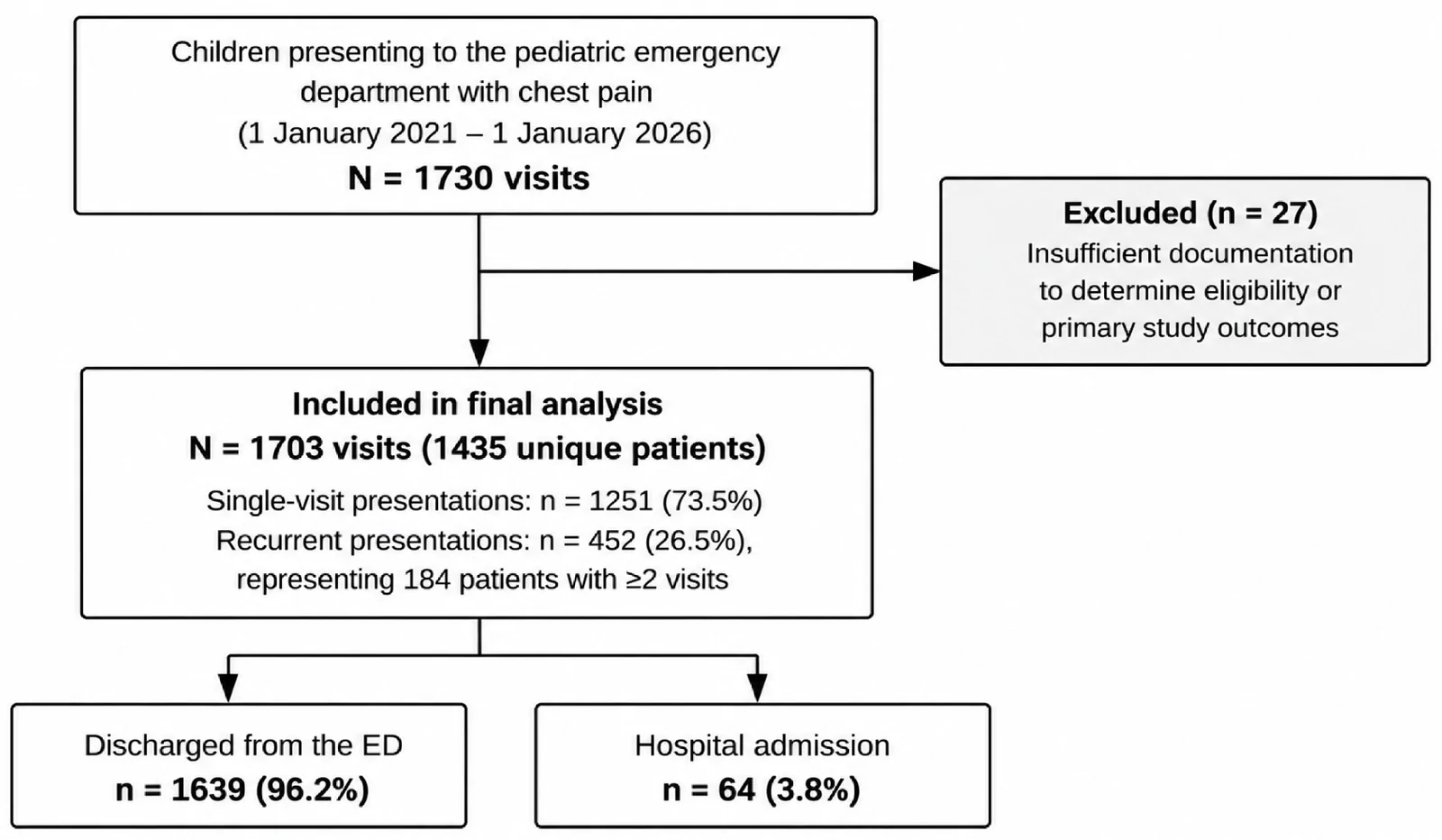
Flow diagram of study participants. ED, emergency department.

The unit of analysis was the ED visit rather than the patient, because presentation, red-flag status, and outcome could differ across successive visits by the same child. Of 1730 eligible visits, 27 were excluded, leaving 1703 visits from 1435 patients; 452 visits (26.5%) by 184 patients were recurrent presentations. Within-patient dependence due to recurrent visits was addressed in a cluster-robust sensitivity analysis (see below) (Figure 1).

### 2.2. Data Collection, Variables, and Definitions

Data were extracted retrospectively from electronic medical records using a standardized case report form that captured demographics, presentation characteristics, accompanying symptoms (palpitations, syncope, dyspnea, fever), medical and family history, vital signs, complete blood count parameters (including NLR and PLR), CRP, and results of ECG, troponin, chest radiography, and echocardiography (obtained at the physician’s discretion), along with cardiology consultation status, admission, and final diagnosis (musculoskeletal, respiratory, gastrointestinal, psychogenic, cardiac, or other). No standardized protocol or predefined criteria were used for chest radiography or echocardiography; these investigations were ordered selectively by the treating physician based on the individual clinical assessment. Each patient’s hospital protocol number linked repeat visits.

A composite red-flag variable, defined a priori, was considered present if any of the following was documented: effort-related chest pain, syncope, palpitations, dyspnea, fever, or a family history of sudden cardiac death. Recurrent presentation was defined at the visit level as an index visit occurring after at least one prior chest-pain visit by the same patient; patients with a single visit were coded as non-recurrent. The primary outcome was hospital admission (ward, pediatric intensive care unit, or ED observation unit); the secondary outcome was a cardiac diagnosis, including myocarditis, pericarditis, or other cardiac pathology identified after pediatric cardiology evaluation. CRP was analyzed both continuously and dichotomized at >5 mg/L, which corresponds to the upper limit of the normal reference range for the assay used in our institutional laboratory. NLR and PLR were analyzed as continuous variables and standardized (per 1-SD increase) in regression models because their raw scales were not comparable.

### 2.3. Statistical Analysis

All analyses were performed in R version 4.6.0 (R Foundation for Statistical Computing, Vienna, Austria); IBM SPSS Statistics version 21.0 (IBM Corp., Armonk, NY, USA) was used only to store and export the source data file. Conformity of empirical distributions to normality was assessed using the Shapiro–Wilk test, supported by visual inspection of histograms and Q–Q plots and by examination of skewness coefficients. All continuous variables deviated significantly from normality on Shapiro–Wilk testing (all *p* < 0.001). Laboratory variables showed marked right skew (skewness 2.7–8.7 for white blood cell count, the neutrophil-to-lymphocyte ratio, the platelet-to-lymphocyte ratio, and C-reactive protein), with visual departure from the reference line concentrated in the upper tail. Age was more nearly symmetric (skewness −0.56) but still deviated significantly from normality, which, in a sample of this size, may reflect the sensitivity of the Shapiro–Wilk test to minor departures. Because no continuous variable met criteria for a normal distribution on combined statistical and visual assessment, none was summarized as mean ± standard deviation; all are presented as median [Q1–Q3] and compared using the Mann–Whitney U test. Categorical variables were compared using the chi-square or Fisher’s exact test, as appropriate.

Independent predictors of hospital admission and cardiac diagnosis were identified using multivariable Firth penalized logistic regression (R package logistf), with 95% CIs derived from the profile penalized likelihood [13,14,15,16], chosen given the low events-per-variable ratio for admission (~6.8). Candidate predictors, selected for clinical relevance and univariable results, were age, sex, chronic disease, red-flag findings, abnormal ECG, CRP > 5 mg/L, NLR, PLR, and recurrent presentation, and were entered identically in both models.

Four sensitivity analyses were conducted for the models: exclusion of CRP, the covariate with the most missing data; stratification by age group; re-estimation with cluster-robust standard errors (CR2 correction), clustered by patient [17]; and the addition of elevated troponin as a covariate to both models. Missing data were handled via complete-case analysis, yielding analytic samples of *n* = 1371 for the primary models, *n* = 1596 for the CRP-free sensitivity model, and *n* = 1338 for the troponin-inclusive sensitivity models. Two-sided *p* < 0.05 was considered significant.

## 3. Results

### 3.1. Patient Characteristics

During the study period, 1730 visits for chest pain were identified as eligible. After excluding 27 visits with insufficient documentation, 1703 visits were included in the final analysis, representing 1435 unique patients. Of these, 452 visits (26.5%) were recurrent presentations by 184 patients who presented on two or more occasions, while the remaining 1251 visits (73.5%) were single presentations. Sixty-four visits (3.8%) resulted in hospital admission, whereas 1639 (96.2%) were discharged. The median age of the cohort was 14.0 years (IQR, 10.0–16.0), and 904 (53.1%) were female.

Baseline characteristics by admission status are shown in Table 1. Admitted patients were more often male (64.1% vs. 46.2%, *p* = 0.007) and more likely to have red-flag findings (71.9% vs. 32.8%, *p* < 0.001), abnormal ECG findings (11.1% vs. 1.8%, *p* < 0.001), and CRP > 5 mg/L (52.5% vs. 19.3%, *p* < 0.001). Admitted patients also had higher NLR (median, 2.6 vs. 1.7; *p* < 0.001) and CRP levels (median, 7.1 vs. 2.0 mg/L; *p* < 0.001) and were more likely to be recurrent presenters (43.8% vs. 25.9%, *p* = 0.002). Age, chronic disease prevalence, and white blood cell count did not differ significantly between groups (all *p* ≥ 0.076).

### 3.2. Etiology of Chest Pain

Musculoskeletal disorders were the leading cause of chest pain (63.8%), followed by respiratory (12.1%), gastrointestinal (8.2%), psychogenic (5.9%), cardiac (5.3%), and other causes (4.6%) (Supplementary Table S3a).

### 3.3. Diagnostic Evaluation

Electrocardiography was performed in 98.8% of visits (abnormal in 2.1%); troponin measurement in 94.8% (elevated in 6.0%); chest radiography in 71.2% (abnormal in 5.9%); and echocardiography in 4.3% (abnormal in 79.7%) (Table 2). The three most frequently ordered tests—ECG, troponin, and chest radiography—had a median utilization of 94.8% but a median diagnostic yield of only 5.9%, in contrast to echocardiography, which was performed selectively (4.3%) yet yielded an abnormal finding in 79.7% of studies. Among chest radiographs, pulmonary infiltration or pneumonia was the most common abnormality (4.8%); among echocardiograms, findings categorized as myocarditis were the most frequently recorded (41/74, 55.4%), followed by valvular abnormalities (17.6%) and pericardial effusion (1.4%) (Supplementary Tables S5 and S6).

### 3.4. Recurrent Presentation

**Figure 2:**
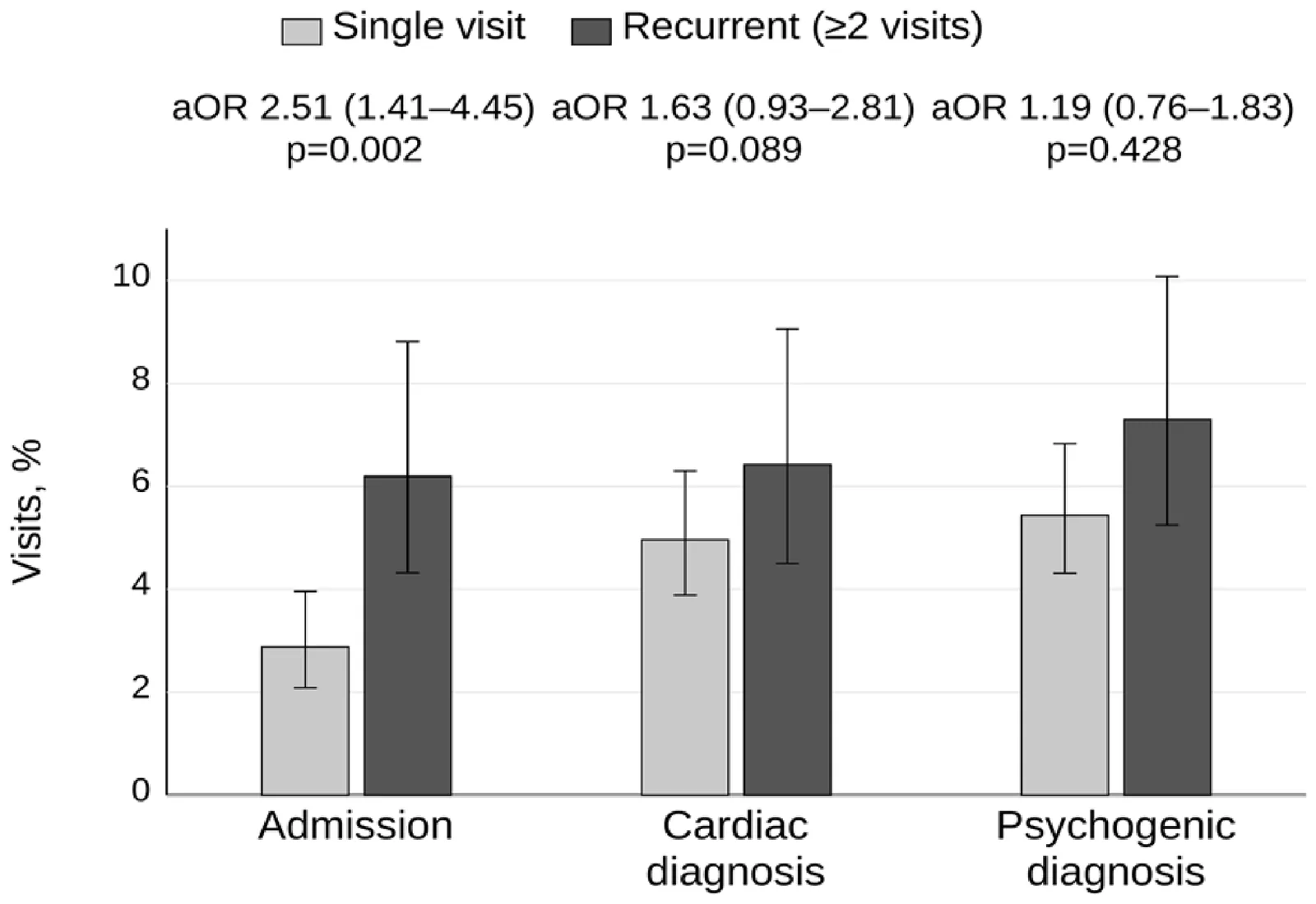
Recurrent presentations and clinical outcomes across single-visit and recurrent visits. Bars show the percentage of visits for each outcome; error bars indicate 95% Wilson confidence intervals for each proportion; aOR, adjusted odds ratio from the multivariable Firth logistic regression model (Table 4).

Because recurrent visits accounted for more than a quarter of the cohort, we examined recurrence as a distinct clinical variable (Table 3, Figure 2). Admission was more frequent in recurrent than in single-visit presentations (6.2% vs. 2.9%), a difference that remained significant after multivariable adjustment (OR, 2.51; 95% CI, 1.41–4.45; *p* = 0.002). Cardiac diagnosis (6.4% vs. 5.0%; adjusted OR, 1.63; 95% CI, 0.93–2.81; *p* = 0.089) and psychogenic diagnosis (7.3% vs. 5.4%; adjusted OR, 1.19; 95% CI, 0.76–1.83; *p* = 0.428) did not differ significantly by recurrence status. Among the 184 patients with recurrent visits, the median interval between the first and last visit was 7.5 months (IQR, 1.3–17.0), and the median interval between consecutive visits was 133 days (IQR, 16–324). In total, 30.6% of recurrent patients returned within 30 days and 42.5% within 90 days of a prior visit. Among admitted patients, the distribution of final diagnoses did not differ significantly between single-visit and recurrent presentations (*p* = 0.051; Supplementary Table S4). However, respiratory diagnoses were more common among recurrent admissions (32.1% vs. 8.3%), and cardiac diagnoses were more common among single-visit admissions (52.8% vs. 35.7%).

### 3.5. Predictors of Hospital Admission

In the multivariable Firth logistic regression model (*n* = 1371), red-flag findings (OR, 5.15; 95% CI, 2.91–9.55), abnormal ECG findings (OR, 4.00; 95% CI, 1.46–10.00), CRP > 5 mg/L (OR, 3.71; 95% CI, 2.07–6.64), male sex (OR, 2.13; 95% CI, 1.22–3.78), and recurrent presentation (see Recurrent presentation, above) were independently associated with hospital admission (all *p* ≤ 0.009); age, chronic disease, NLR, and PLR were not (Table 4). These associations were essentially unchanged after accounting for within-patient clustering with cluster-robust standard errors, including for recurrent presentation (OR, 2.56; 95% CI, 1.34–4.89; *p* = 0.005; Supplementary Table S1). Excluding CRP from the model (*n* = 1596) did not materially change these associations, and higher NLR emerged as an additional independent predictor (OR, 1.36; 95% CI, 1.05–1.79; *p* = 0.020; Supplementary Table S2).

### 3.6. Predictors of Cardiac Diagnosis

In the multivariable Firth logistic regression model for cardiac diagnosis (*n* = 1371), abnormal ECG findings (OR, 27.70; 95% CI, 11.45–71.25), CRP > 5 mg/L (OR, 4.56; 95% CI, 2.61–8.00), red-flag findings (OR, 3.05; 95% CI, 1.83–5.16), male sex (OR, 1.95; 95% CI, 1.16–3.33), and increasing age (OR, 1.15 per year; 95% CI, 1.06–1.25) were independently associated with cardiac diagnosis (all *p* ≤ 0.012); chronic disease, NLR, and PLR were not (Table 4). In contrast to its association with hospital admission, recurrent presentation was not independently associated with cardiac diagnosis (OR, 1.63; 95% CI, 0.93–2.81; *p* = 0.089). Troponin elevation was strongly associated with a cardiac diagnosis: it was present in 66 of 89 (74.2%) children with a cardiac diagnosis versus 31 of 1523 (2.0%) without (χ^2^ = 760.7, *p* < 0.001). However, in the multivariable model, the estimate was affected by quasi-separation (OR, 205.8; 95% CI, 91.6–519.2), with attenuation of other estimates, particularly for CRP > 5 mg/L (OR, 4.56 to 1.47) (Supplementary Table S7). Because troponin results were available during pediatric cardiology confirmation of cardiac diagnoses, troponin was not retained in the primary models and is discussed further in the Discussion.

### 3.7. Combined Effect of CRP and Red-Flag Findings

Hospital admission and cardiac diagnosis rates differed significantly across CRP/red-flag subgroups (Table 5). The lowest rates were observed among visits with neither finding (1.4% and 2.2%, respectively), whereas the highest rates were observed among visits with both elevated CRP and red-flag findings (22.2% and 24.8%, respectively) (both *p* < 0.001, Pearson chi-square test).

### 3.8. Sensitivity Analyses

Hospital admission rates did not differ significantly across age groups (0–5 years, 0.0%; 6–11 years, 2.7%; 12–18 years, 4.4%; *p* = 0.098). However, cardiac diagnosis increased across age groups (0.0%, 3.4%, and 6.5%; *p* = 0.008), while psychogenic diagnosis differed significantly by age group (4.2%, 2.9%, and 7.4%; *p* = 0.0005); the proportion of recurrent presentations also increased with age (6.2%, 23.7%, and 28.7%) (Supplementary Table S3b). Regression analysis could not be performed in the youngest age group because no admissions or cardiac diagnoses occurred in this subgroup.

## 4. Discussion

In this cohort of 1703 children with chest pain, only 3.8% required admission and 5.3% had a cardiac diagnosis, confirming that pediatric chest pain is overwhelmingly benign. Musculoskeletal and idiopathic etiologies predominated, consistent with previous studies [1,2]. Our cardiac diagnosis rate falls within the range reported by comparable cohorts, from 0.6% among 4288 patients [4] to 4% in an early series of 407 children [18]. These studies similarly identified musculoskeletal and idiopathic causes as leading etiologies [4,18,19]. A more recent cohort of over 10,000 encounters reported a comparably low cardiac rate of 0.7–1.1% [20], suggesting this pattern has remained stable despite evolving testing practices.

Male sex, red-flag findings, an abnormal ECG, and CRP > 5 mg/L were independently associated with admission and/or cardiac diagnosis; increasing age was independently associated with cardiac diagnosis, despite no significant univariable association with admission in Table 1 (*p* = 0.076), likely reflecting confounding by red-flag findings and abnormal ECG, both of which are age-related. The prognostic value of red-flag findings corroborates previous work [7,8], though the male-sex association should be interpreted cautiously: absent a clear mechanistic explanation, it may reflect differences in etiology distribution rather than a direct biological effect and remains speculative pending replication. The strong association between abnormal ECG and cardiac diagnosis (OR, 27.70) reaffirms its central role, despite a low overall abnormality rate (2.1%), consistent with the limited diagnostic yield of screening ECG reported previously [1]. This combination of low overall yield but strong association with cardiac diagnosis supports selective ECG use guided by clinical findings.

One of the most clinically relevant findings was the independent association of elevated CRP (>5 mg/L) with both admission and cardiac diagnosis, even after adjusting for recurrent presentation. This is biologically plausible: CRP has shown strong discriminative performance in pediatric myocarditis (AUC, 0.85) [21], and composite inflammatory indices independently predict a fulminant disease course [22]. Among the 74 patients who underwent echocardiography, findings categorized as myocarditis were recorded in 41 (55.4%); however, these echocardiographic findings should not be interpreted as a descriptive breakdown of the 91 patients classified as having a cardiac diagnosis. Combined with red-flag findings, elevated CRP was associated with markedly higher event rates, increasing from 1.4%/2.2% to 22.2%/24.8%. However, given the retrospective design and selective CRP testing, these findings should not be interpreted as supporting universal CRP testing in children with chest pain. The potential value of this combination for risk stratification requires prospective validation. In contrast, NLR and PLR were not independently associated with either outcome in the CRP-inclusive models; NLR reached significance only when CRP was excluded, likely reflecting collinearity among overlapping inflammatory signals. This contrasts with reports in other pediatric conditions—asthma, pneumonia, trauma—where NLR and PLR show independent prognostic value [9,10], suggesting their utility may be condition-specific.

A novel finding was the independent association between recurrent presentation and admission: children with a prior chest-pain visit had more than twice the odds of admission (OR, 2.51; 95% CI, 1.41–4.45), an association that persisted after cluster-robust adjustment (OR, 2.56; 95% CI, 1.34–4.89), confirming that it was not an artifact of treating repeat visits as separate observations. Contrary to our initial hypothesis that recurrent chest pain reflects an underlying anxiety or somatic pattern, recurrence was not associated with either a psychogenic (OR, 1.19; 95% CI, 0.76–1.83) or a cardiac diagnosis (OR, 1.63; 95% CI, 0.93–2.81). This suggests that recurrent presentations may reflect factors beyond greater disease severity, including unresolved symptoms, parental concern or anxiety, differences in access to care or referral patterns, and a lower clinician threshold for admission among children with repeated presentations. Recurrent symptoms have been reported in approximately one-third of children evaluated for chest pain [11], and broader pediatric ED data indicate that recurrent utilization is associated with chronic disease burden and healthcare use patterns [12]. That cardiac and psychogenic diagnoses—but not recurrence—increased with age is compatible with evidence linking non-cardiac chest pain to anxiety disorders [23], suggesting that age, rather than recurrence, is the more relevant psychogenic marker. This null finding nonetheless contrasts with adult evidence that depression independently predicts 30-day recurrent chest pain (OR, 2.11; 95% CI, 1.18–3.79) [24], possibly reflecting our reliance on discharge codes rather than formal psychiatric screening, which likely underestimates any psychogenic contribution to recurrence, a limitation discussed below.

Diagnostic testing patterns mirror prior concerns about the limited yield of routine cardiac testing in children with chest pain [1]. Similar patterns have been reported elsewhere: in a Portuguese pediatric ED, ECG and chest radiography were obtained in more than half of visits, while cardiac biomarkers were requested in only 8.9% [25]; in a Singaporean cohort, cardiac enzyme testing rose from virtually none to 26.1% of visits without a corresponding increase in cardiac diagnoses [26]. In our cohort, ECG and troponin were performed in most patients, yet abnormal findings were uncommon (2.1% and 6.0%), consistent with the limited troponin yield reported in an unselected pediatric ED population [27]. Echocardiography, by contrast, was performed selectively (4.3%) yet showed a substantially higher yield (79.7%); this reflects appropriate clinician-directed selection of higher-risk patients rather than echocardiography itself being a high-value screening test—a form of indication bias inherent in selectively ordered testing. Together, these findings across multiple pediatric ED cohorts [25,26] suggest that broader testing does not consistently translate into higher yield, supporting the reservation of resource-intensive investigations for children at higher clinical risk, a concept aligned with previously described standardized assessment and management pathways for pediatric chest pain [5,28,29].

This study’s strengths include a large, consecutive cohort spanning five years, evaluation of two clinically relevant outcomes, Firth penalized regression to reduce small-sample bias [13,14,15,16], and robustness confirmed across multiple sensitivity analyses, including a cluster-robust adjustment for within-patient dependence due to recurrent visits [17].

Limitations include the retrospective, single-center design, which may limit generalizability and introduce documentation bias; hospital admission was a clinician-determined outcome and therefore reflects both underlying disease severity and physician decision-making. Because red-flag findings, abnormal ECG results, and elevated CRP may have influenced the decision to admit, their associations with admission may partly reflect this inherent circularity and should not be interpreted solely as independent markers of disease severity. ECG findings were recorded only as normal or abnormal, without systematic documentation of specific abnormality types, precluding reliable subclassification; the individual components of the composite red-flag variable were not evaluated separately in the multivariable models; because the composite included dyspnea and fever alongside more cardiac-specific features, its association with admission may partly reflect overall illness severity rather than cardiac risk alone; and specific cardiac diagnostic subtypes were not systematically recorded, preventing a detailed descriptive breakdown of cardiac diagnoses. All patients with a cardiac diagnosis underwent pediatric cardiology consultation; however, cardiology evaluation was not performed uniformly across the cohort. Therefore, differential referral for cardiology consultation may have introduced verification bias, potentially leading to underrecognition of cardiac disease among non-consulted patients. Because cardiac diagnoses were confirmed by pediatric cardiology consultation, the investigations available to the consulting cardiologist are not fully independent of the outcome. This dependency was most pronounced for troponin, which was elevated in 74.2% of children with a cardiac diagnosis and produced a quasi-separated, clinically uninterpretable estimate when entered into the multivariable model. Abnormal ECG behaved differently: it was absent in 66 of 90 children (73.3%) ultimately diagnosed with cardiac disease, and its estimate remained within an interpretable range, indicating that ECG was neither necessary nor sufficient for the diagnosis. We therefore retained ECG in the primary models while presenting troponin-inclusive models as a sensitivity analysis. Nonetheless, both associations should be interpreted as reflecting the diagnostic pathway rather than as independent causal predictors. CRP and complete blood count testing were incomplete, performed at the discretion of the clinician, and addressed using a complete-case analysis. Because CRP was not measured at all visits and was likely obtained selectively in children with greater clinical concern, selection bias cannot be excluded, and the observed associations with CRP may not be generalizable to all children presenting with chest pain. Other limitations include the selective use of echocardiography, precluding assessment of its performance across the full cohort; recurrent presentations captured only in our institutional records, which may underestimate true recurrence; and follow-up limited to our institution, as noted in a prior follow-up study [11]. Psychogenic diagnoses were based on discharge codes rather than standardized psychiatric assessment, which may have underestimated the contribution of underlying psychiatric disorders. Structured psychiatric assessment has identified psychiatric disorders in a substantially higher proportion of children with non-cardiac chest pain than the 5.9% psychogenic diagnosis rate observed in our cohort [23], underscoring the need for prospective studies incorporating formal psychiatric evaluation.

## 5. Conclusions

Chest pain in children presenting to the ED was predominantly benign in this cohort, whereas red-flag findings, an abnormal ECG, elevated CRP, and recurrent presentations were associated with a higher likelihood of hospital admission. Cardiac and psychogenic diagnoses were not associated with recurrence; cardiac diagnosis increased with age, whereas psychogenic diagnosis differed across age groups. The combination of elevated CRP and red-flag findings was associated with higher rates of admission and cardiac diagnosis, suggesting potential value for risk stratification that requires prospective validation. The low yield of routine cardiac testing, contrasted with the high yield of selective echocardiography, further supports a risk-stratified rather than routine diagnostic approach. Prospective multicenter studies are warranted before incorporation into routine practice.

## Figures and Tables

**Table 1:** Baseline characteristics of study visits according to hospital admission status (N = 1703).

Variable	Overall (N = 1703)	Not Admitted (N = 1639)	Admitted (N = 64)	*p*-Value
Age, years, median (IQR)	14.0 (10.0–16.0)	14.0 (10.0–16.0)	15.0 (12.0–16.0)	0.076
Sex, *n* (%)				0.007
Female	904 (53.1%)	881 (53.8%)	23 (35.9%)	
Male	799 (46.9%)	758 (46.2%)	41 (64.1%)	
Chronic disease, *n* (%)	522 (30.6%)	500 (30.5%)	22 (34.4%)	0.5
Red-flag finding, *n* (%)				<0.001
Absent	1120 (65.8%)	1102 (67.2%)	18 (28.1%)	
Present	583 (34.2%)	537 (32.8%)	46 (71.9%)	
Abnormal ECG, *n* (%) ^a^	36 (2.1%)	29 (1.8%)	7 (11.1%)	<0.001
CRP > 5 mg/L, *n* (%) ^b^	288 (20.8%)	256 (19.3%)	32 (52.5%)	<0.001
WBC, /µL, median (IQR) ^c^	8670 (7200–10,550)	8670 (7200–10,540)	8980 (7270–10,775)	0.6
NLR, median (IQR) ^c^	1.7 (1.2–2.7)	1.7 (1.2–2.6)	2.6 (1.4–4.3)	<0.001
CRP, mg/L, median (IQR) ^b^	2.0 (2.0–3.7)	2.0 (2.0–3.4)	7.1 (2.0–48.4)	<0.001
Recurrent presentation, *n* (%)				0.002
Single visit	1251 (73.5%)	1215 (74.1%)	36 (56.3%)	
Recurrent (≥2 visits)	452 (26.5%)	424 (25.9%)	28 (43.8%)	

Data are presented as *n* (%) unless otherwise indicated. Continuous variables are presented as median (interquartile range [IQR]). Categorical variables were compared using the chi-square test or Fisher’s exact test, as appropriate. Continuous variables were compared using the Mann–Whitney U test. CRP, C-reactive protein; ECG, electrocardiography; IQR, interquartile range; NLR, neutrophil-to-lymphocyte ratio. ^a^ 21 missing; ^b^ 317–318 missing (CRP-based variables); ^c^ 96 missing (complete blood count-based variables).

**Table 2:** Utilization and diagnostic yield of ancillary tests.

Test	Utilization, *n*/N (%)	Diagnostic Yield, *n*/N (%)
ECG	1682/1703 (98.8%)	36/1682 (2.1%)
Troponin	1615/1703 (94.8%)	97/1612 (6.0%)
Chest radiograph (CXR)	1212/1703 (71.2%)	71/1212 (5.9%)
Echocardiography	74/1703 (4.3%)	59/74 (79.7%)

Diagnostic yield is calculated for tests with a documented result. The utilization and yield denominators are identical for ECG, chest radiography, and echocardiography. For troponin, three ordered tests lacked a documented result, so the yield denominator (1612) is slightly smaller than the utilization denominator (1615); this difference (0.2% of tests performed) does not materially affect the reported yield. ECG, electrocardiography; CXR, chest radiograph.

**Table 3:** Recurrent presentation in relation to clinical outcomes and visit timing. (**A**) Recurrent presentation and clinical outcomes. (**B**) Timing characteristics of recurrent visits (*n* = 184 patients).

**(A)**				
**Outcome**	**Single Visit, *n* (%)**	**Recurrent, *n* (%)**	**Adjusted OR (95% CI)**	***p***
Admission	36 (2.9%)	28 (6.2%)	2.51 (1.41–4.45)	0.002
Cardiac diagnosis	62 (5.0%)	29 (6.4%)	1.63 (0.93–2.81)	0.089
Psychogenic diagnosis	68 (5.4%)	33 (7.3%)	1.19 (0.76–1.83)	0.428
**(B)**				
**Measure**			**Value**	
First-to-last visit interval, months, median [IQR]			7.5 [1.3–17.0]	
Interval between consecutive visits, days, median [IQR]			133 [16–324]	
Repeat visit within 30 days, %			30.6	
Repeat visit within 90 days, %			42.5	

The adjusted ORs for hospital admission and cardiac diagnosis were derived from the full multivariable Firth logistic regression models (Table 4; N = 1371). The adjusted OR for psychogenic diagnosis was derived from a model adjusted for age, sex, and chronic disease. Unadjusted comparisons were performed using the chi-square test. IQR, interquartile range; OR, odds ratio; CI, confidence interval.

**Table 4:** Multivariable Firth logistic regression models for hospital admission and cardiac diagnosis.

Variable	Admission OR (95% CI)	*p*	Cardiac Diagnosis OR (95% CI)	*p*
Age, years	1.05 (0.97–1.14)	0.256	1.15 (1.06–1.25)	<0.001
Sex (male vs. female)	2.13 (1.22–3.78)	0.007	1.95 (1.16–3.33)	0.012
Chronic disease (yes vs. no)	1.08 (0.60–1.91)	0.789	0.93 (0.53–1.60)	0.798
Red-flag finding (yes vs. no)	5.15 (2.91–9.55)	<0.001	3.05 (1.83–5.16)	<0.001
Abnormal ECG (yes vs. no)	4.00 (1.46–10.00)	0.009	27.70 (11.45–71.25)	<0.001
CRP > 5 mg/L (yes vs. no)	3.71 (2.07–6.64)	<0.001	4.56 (2.61–8.00)	<0.001
NLR (per 1-SD increase)	1.09 (0.77–1.48)	0.608	0.99 (0.68–1.38)	0.939
PLR (per 1-SD increase)	1.01 (0.65–1.39)	0.951	0.89 (0.54–1.39)	0.660
Recurrent presentation (yes vs. no)	2.51 (1.41–4.45)	0.002	1.63 (0.93–2.81)	0.089

OR, odds ratio; CI, confidence interval; CRP, C-reactive protein; ECG, electrocardiography; NLR, neutrophil-to-lymphocyte ratio; PLR, platelet-to-lymphocyte ratio. Multivariable models were fit using Firth penalized logistic regression, and 95% CIs were derived from the profile penalized likelihood. Both models were restricted to visits with complete data for all covariates (N = 1371). The admission model included 61 events, and the cardiac diagnosis model included 80 events. NLR and PLR are reported per 1-SD increase.

**Table 5:** Combined effect of elevated CRP and red-flag findings on admission and cardiac diagnosis.

Subgroup	N	Admission, *n* (%)	Cardiac Diagnosis, *n* (%)
CRP(−)/Red flag(−)	724	10 (1.4%)	16 (2.2%)
CRP(−)/Red flag(+)	374	19 (5.1%)	21 (5.6%)
CRP(+)/Red flag(−)	171	6 (3.5%)	14 (8.2%)
CRP(+)/Red flag(+)	117	26 (22.2%)	29 (24.8%)

Comparisons across subgroups were performed using the Pearson chi-square test (df = 3): χ^2^ = 104.74, *p* < 0.001 for hospital admission and χ^2^ = 96.52, *p* < 0.001 for cardiac diagnosis. The analysis was restricted to visits with complete CRP data (N = 1386). CRP, C-reactive protein.

## Data Availability

The data presented in this study are available upon reasonable request from the corresponding author. The data are not publicly available due to privacy and ethical restrictions on patient-level clinical data.

## Supplementary Materials

The following supporting information can be downloaded at: https://www.mdpi.com/article/10.3390/medicina62091809/s1, Table S1: Cluster-robust sensitivity analysis for the hospital admission model; Table S2: Sensitivity analysis for the hospital admission model excluding CRP; Table S3a: Distribution of final diagnoses (etiology of chest pain), N = 1703; Table S3b: Age-group sensitivity analysis: admission, cardiac diagnosis, psychogenic diagnosis, and recurrent presentation; Table S4: Distribution of final diagnoses among admitted visits: single vs. recurrent presentation (N = 64); Table S5: Chest radiograph findings among studies performed (N = 1212); Table S6: Echocardiographic findings among studies performed (N = 74); Table S7: Sensitivity analysis adding elevated troponin to the multivariable models for hospital admission and cardiac diagnosis.

## References

[1] Huang S.W., Liu Y.K. (2024). Pediatric chest pain: A review of diagnostic tools in the pediatric emergency department. Diagnostics.

[2] Alsabri M., Elshanbary A.A., Nourelden A.Z., Fathallah A.H., Zaazouee M.S., Pincay J., Nakadar Z., Wasem M., Aeder L. (2024). Chest pain in pediatric patients in the emergency department—Presentation, risk factors, and outcomes—A systematic review and meta-analysis. PLoS ONE.

[3] Fogliazza F., Cifaldi M., Antoniol G., Canducci N., Esposito S. (2024). Approaches to pediatric chest pain: A narrative review. J. Clin. Med..

[4] Drossner D.M., Hirsh D.A., Sturm J.J., Mahle W.T., Goo D.J., Massey R., Simon H.K. (2011). Cardiac disease in pediatric patients presenting to a pediatric ED with chest pain. Am. J. Emerg. Med..

[5] Mohan S., Nandi D., Stephens P., McFarrej M., Vogel R.L., Bonafide C.P. (2018). Implementation of a clinical pathway for chest pain in a pediatric emergency department. Pediatr. Emerg. Care.

[6] Jakubiak G.K., Pawlas N., Starzak M., Stanek A., Cieślar G. (2026). Associations of High-Sensitivity Cardiac Troponin T, D-Dimer, and N-Terminal Pro–B-Type Natriuretic Peptide with Subclinical Cardiovascular Dysfunction in the General Population: A Retrospective Cross-Sectional Study. Vasc. Health Risk Manag..

[7] Friedman K.G., Alexander M.E. (2013). Chest pain and syncope in children: A practical approach to the diagnosis of cardiac disease. J. Pediatr..

[8] Saleeb S.F., Li W.Y., Warren S.Z., Lock J.E. (2011). Effectiveness of screening for life-threatening chest pain in children. Pediatrics.

[9] Xu M., Zhou L., Zhang J., Luo S., Zhao Y., Xiong W. (2023). Neutrophil to lymphocyte ratio in pediatric patients with asthmatic exacerbation and community-acquired pneumonia. BMC Pediatr..

[10] Tekin Y.K. (2019). Are neutrophil-to-lymphocyte and platelet-to-lymphocyte ratios associated with mortality in pediatric trauma patients? A retrospective study. Rambam Maimonides Med. J..

[11] Gesuete V., Fregolent D., Contorno S., Tamaro G., Barbi E., Cozzi G. (2020). Follow-up study of patients admitted to the pediatric emergency department for chest pain. Eur. J. Pediatr..

[12] Vrijlandt S.E.W., Nieboer D., Zachariasse J.M., Oostenbrink R. (2022). Characteristics of pediatric emergency department frequent visitors and their risk of a return visit: A large observational study using electronic health record data. PLoS ONE.

[13] Firth D. (1993). Bias reduction of maximum likelihood estimates. Biometrika.

[14] Heinze G., Schemper M. (2002). A solution to the problem of separation in logistic regression. Stat. Med..

[15] Uno S., Noma H., Gosho M. (2024). Firth-type penalized methods of the modified Poisson and least-squares regression analyses for binary outcomes. Biom. J..

[16] Gosho M., Ohigashi T., Nagashima K., Ito Y., Maruo K. (2023). Bias in odds ratios from logistic regression methods with sparse data sets. J. Epidemiol..

[17] Pustejovsky J.E., Tipton E. (2018). Small-sample methods for cluster-robust variance estimation and hypothesis testing in fixed effects models. J. Bus. Econ. Stat..

[18] Selbst S.M., Ruddy R.M., Clark B.J., Henretig F.M., Santulli T. (1988). Pediatric chest pain: A prospective study. Pediatrics.

[19] Rowe B.H., Dulberg C.S., Peterson R.G., Vlad P., Li M.M. (1990). Characteristics of children presenting with chest pain to a pediatric emergency department. CMAJ Can. Med. Assoc. J..

[20] Atmanli A., Yen K., Zhou A.Z. (2024). Diagnostic testing for chest pain in a pediatric emergency department and rates of cardiac disease before and during the COVID-19 pandemic: A retrospective study. Front. Pediatr..

[21] Yapar Gümüş C., Yurdakul Ertürk E., Kaşko Arıcı Y., Altınok E.İ., Bayrak T., Bayrak A., Kasar T. (2026). Ischemia-modified albumin in children with clinically suspected acute myocarditis: Diagnostic performance and incremental value beyond conventional biomarkers. Front. Pediatr..

[22] Yaradilmiş R.M., Güneylioğlu M.M., Öztürk B., Göktüğ A., Aydın O., Güngör A., Bodur İ., Kaya Ö., Örün U.A., Karacan C.D. (2023). A novel marker for predicting fulminant myocarditis: Systemic immune-inflammation index. Pediatr. Cardiol..

[23] Bozatlı L., Deveci M., Görker I. (2025). Anxiety disorders in children with non-cardiac chest pain: Is routine screening needed in pediatric clinics?. Pediatr. Int..

[24] Kim Y., Soffler M., Paradise S., Jelani Q.U., Dziura J., Sinha R., Safdar B. (2017). Depression is associated with recurrent chest pain with or without coronary artery disease: A prospective cohort study in the emergency department. Am. Heart J..

[25] Pissarra R., Pereira M., Amorim R., Neto B.P., Lourenço L., Santos L.A. (2022). Chest pain in a pediatric emergency department: Clinical assessment and management reality in a third-level Portuguese hospital. Porto Biomed. J..

[26] Tan J.Z., Zhang D.Z., Sundararaghavan S., Ganapathy S., Choo J.T.L., Rajendram M.F., Chong S.L. (2023). Chest pain attendances to a paediatric emergency department pre- and post-COVID-19 vaccination. Transl. Pediatr..

[27] Brancato F., De Rosa G., Gambacorta A., Nunziata A., Ferrara P., Buonsenso D., Covino M., Chiaretti A. (2021). Role of troponin determination to diagnose chest pain in the pediatric emergency department. Pediatr. Emerg. Care.

[28] Verghese G.R., Friedman K.G., Rathod R.H., Meiri A., Saleeb S.F., Graham D.A., Geggel R.L., Fulton D.R. (2012). Resource utilization reduction for evaluation of chest pain in pediatrics using a novel standardized clinical assessment and management plan (SCAMP). J. Am. Heart Assoc..

[29] Kane D.A., Friedman K.G., Fulton D.R., Geggel R.L., Saleeb S.F. (2016). Needles in hay II: Detecting cardiac pathology by the pediatric chest pain standardized clinical assessment and management plan. Congenit. Heart Dis..

