# Supplementary material for: Predictors of Hospital Admission and Cardiac Diagnosis in Children Presenting with Chest Pain: The Role of Recurrent Presentation and Inflammatory Markers
Source: Medicina (Kaunas). 2026 Sep 19;62(9):1809. doi: 10.3390/medicina62091809 (PMC13609831; doi:10.3390/medicina62091809)
Supplement: Supplementary file 1 [file medicina-62-01809-s001.zip › medicina-4521278-supplementary.pdf]

Supplementary Tables

Supplementary Table S1. Cluster-robust sensitivity analysis for the hospital admission model

Variable	Main model (Firth) OR (95% CI)	p	Cluster-robust OR (95% CI)	p (cluster -robust)
Age, years	1.05 (0.97–1.14)	0.256	1.05 (0.97–1.14)	0.210
Sex (male vs. female)	2.13 (1.22–3.78)	0.007	2.18 (1.19–3.98)	0.012
Chronic disease (yes vs. no)	1.08 (0.60–1.91)	0.789	1.07 (0.59–1.93)	0.822
Red-flag finding (yes vs. no)	5.15 (2.91–9.55)	<0.001	5.37 (2.98–9.69)	<0.001
Abnormal ECG (yes vs. no)	4.00 (1.46–10.00)	0.009	3.96 (1.45–10.78)	0.012
CRP >5 mg/L (yes vs. no)	3.71 (2.07–6.64)	<0.001	3.86 (2.10–7.09)	<0.001
NLR (per 1-SD increase)	1.09 (0.77–1.48)	0.608	1.09 (0.75–1.58)	0.666
PLR (per 1-SD increase)	1.01 (0.65–1.39)	0.951	0.96 (0.66–1.40)	0.828
Recurrent presentation (yes vs. no)	2.51 (1.41–4.45)	0.002	2.56 (1.34–4.89)	0.005

Presented as a robustness check for Table 4 (main text). Cluster-robust standard errors (CR2 correction) were clustered by patient to account for within-patient dependence arising from recurrent visits. Estimates are consistent in direction and magnitude between the Firth and cluster-robust approaches, including for the recurrent-presentation predictor.

Supplementary Table S2. Sensitivity analysis for the hospital admission model excluding CRP

Variable	Main model (CRP included) OR (95% CI), N = 1371	p	Sensitivity model (CRP excluded) OR (95% CI), N = 1596	p
Age, years	1.05 (0.97–1.14)	0.256	1.03 (0.96–1.12)	0.433
Sex (male vs. female)	2.13 (1.22–3.78)	0.007	2.17 (1.28–3.76)	0.004
Chronic disease (yes vs. no)	1.08 (0.60–1.91)	0.789	1.02 (0.58–1.76)	0.948
Red-flag finding (yes vs. no)	5.15 (2.91–9.55)	<0.001	5.01 (2.90–9.06)	<0.001
Abnormal ECG (yes vs. no)	4.00 (1.46–10.00)	0.009	4.99 (1.91–11.79)	0.002
CRP >5 mg/L (yes vs. no)	3.71 (2.07–6.64)	<0.001	—	—
NLR (per 1-SD increase)	1.09 (0.77–1.48)	0.608	1.36 (1.05–1.79)	0.020
PLR (per 1-SD increase)	1.01 (0.65–1.39)	0.951	0.93 (0.61–1.18)	0.644
Recurrent presentation (yes vs. no)	2.51 (1.41–4.45)	0.002	2.27 (1.32–3.90)	0.003

OR: odds ratio; CI: confidence interval. CRP had the greatest amount of missing data (317–318 visits; 18.6–18.7%); excluding it from the model allowed inclusion of an additional 225 visits. All other associations remained materially unchanged; NLR became an independent predictor once CRP was removed, consistent with collinearity between these inflammatory markers.

Supplementary Table S3a. Distribution of final diagnoses (etiology of chest pain), N = 1703

Final diagnosis	n (%)
Musculoskeletal	1087 (63.8%)
Respiratory	206 (12.1%)
Gastrointestinal	140 (8.2%)
Psychogenic	101 (5.9%)
Cardiac	91 (5.3%)

Final diagnosis	n (%)
Other	78 (4.6%)

Supplementary Table S3b. Age-group sensitivity analysis: admission, cardiac diagnosis, psychogenic diagnosis, and recurrent presentation

Age group years	N	Admission, n (%)	Cardiac diagnosis, n (%)	Psychogenic diagnosis, n (%)	Recurrent presentation, n (%)
0–5	48	0 (0.0%)	0 (0.0%)	2 (4.2%)	3 (6.2%)
6–11	524	14 (2.7%)	18 (3.4%)	15 (2.9%)	124 (23.7%)
12–18	1131	50 (4.4%)	73 (6.5%)	84 (7.4%)	325 (28.7%)

Fisher's exact test: p = 0.098 for admission, p = 0.0084 for cardiac diagnosis, p = 0.0005 for psychogenic diagnosis, by age group. A regression model could not be fitted within the 0–5-year subgroup (N = 48) owing to zero admission and cardiac-diagnosis events. Presented as a sensitivity analysis supporting the main multivariable models (Table 4, main text).

Supplementary Table S4. Distribution of final diagnoses among admitted visits: single vs. recurrent presentation (N = 64)

Final diagnosis	Overall (N = 64)	Single visit (N = 36)	Recurrent (N = 28)
Musculoskeletal	13 (20.3%)	9 (25.0%)	4 (14.3%)
Respiratory	12 (18.8%)	3 (8.3%)	9 (32.1%)
Gastrointestinal	0 (0.0%)	0 (0.0%)	0 (0.0%)
Psychogenic	2 (3.1%)	0 (0.0%)	2 (7.1%)
Cardiac	29 (45.3%)	19 (52.8%)	10 (35.7%)
Other	8 (12.5%)	5 (13.9%)	3 (10.7%)

n (%). Fisher's exact test, p = 0.051. Denominators correspond to the admitted-visit subgroup (N = 64: 36 single-visit, 28 recurrent) and were verified against the primary dataset. Interpret with caution given the small cell counts.

Supplementary Table S5. Chest radiograph findings among studies performed (N = 1212)

Radiographic finding	n	%
Normal	1141	94.1
Infiltration / pneumonia	58	4.8
Pneumothorax	8	0.7
Pleural effusion	3	0.2
Other	2	0.2

Related to Table 2 (main text): chest radiograph utilization and overall diagnostic yield.

Supplementary Table S6. Echocardiographic findings among studies performed (N = 74)

Echocardiographic finding	n	%
Normal	15	20.3
Pericardial effusion	1	1.4
Myocarditis	41	55.4
Valvular pathology	13	17.6
Other	4	5.4

Related to Table 2 (main text): echocardiography utilization and overall diagnostic yield.

Table S7. Sensitivity analysis adding elevated troponin to the multivariable models for hospital admission and cardiac diagnosis.

Variable	Admission OR (95% CI)	p	Cardiac diagnosis OR (95% CI)	p
Age, years	1.00 (0.91-1.09)	0.970	1.16 (1.03-1.31)	0.015
Sex (male vs. female)	1.87 (1.01-3.55)	0.047	1.32 (0.62-2.82)	0.466
Chronic disease (yes vs. no)	1.45 (0.75-2.79)	0.263	2.14 (0.95-5.00)	0.068
Red-flag finding (yes vs. no)	4.98 (2.67-9.77)	<0.001	3.19 (1.53-6.96)	0.002
Abnormal ECG (yes vs. no)	1.27 (0.39-3.80)	0.678	41.99 (11.33-153.75)	<0.001
CRP >5 mg/L (yes vs. no)	2.17 (1.10-4.20)	0.025	1.47 (0.63-3.33)	0.369
NLR (per 1-SD increase)	1.17 (0.79-1.63)	0.410	1.00 (0.54-1.64)	0.989
PLR (per 1-SD increase)	1.06 (0.71-1.46)	0.737	1.30 (0.79-1.96)	0.241
Recurrent presentation (yes vs. no)	3.03 (1.60-5.78)	<0.001	1.98 (0.89-4.45)	0.095
Elevated troponin	15.28 (7.80-30.22)	<0.001	205.81 (91.55-519.21)	<0.001

Sensitivity analysis adding elevated troponin to the primary multivariable models (Firth penalized logistic regression; N = 1338; 59 admission events, 80 cardiac-diagnosis events). The odds ratio for elevated troponin in the cardiac-diagnosis model (205.8; 95% CI 91.6-519.2) indicates quasi-separation and is not interpretable as an independent effect estimate: cardiac diagnoses were confirmed by pediatric cardiology consultation, during which troponin results were available. Inclusion of troponin also attenuated the association of CRP with cardiac diagnosis (OR 4.56 to 1.47) and of abnormal ECG with admission (OR 4.00 to 1.27), consistent with overlapping diagnostic signal.
